# Nurses’ knowledge, attitudes, and willingness to practice hospice care: An analysis of influencing factors

**DOI:** 10.1371/journal.pone.0259647

**Published:** 2022-02-24

**Authors:** Lin Chen, Xiao-Hong Li, Xiao Pan, Qi-Ni Pan, Hui-Qiao Huang, Pin-Yue Tao, Gao-Ye Li, Jin-Hui Ma, Jing-Can Huang

**Affiliations:** 1 Department of Nursing, The Second Affiliated Hospital of Guangxi Medical University, Nanning, Guangxi, China; 2 Department of Nursing, The First Affiliated Hospital of Guangxi Medical University, Nanning, Guxngxi, China; 3 Department of Nursing, The Fifth People’s Hospital of Chengdu, Chengdu, Sichuan, China; 4 Department of Nursing, The Affiliated Hospital of Guilin Medical University, Guilin, Guangxi, China; Sunway University, MALAYSIA

## Abstract

**Background:**

Hospice care is a multidisciplinary approach that focused on patients’ quality of life, and nurses allocate more of their time with patients and patients’ families than those nurses working in other disciplines. Nurses’ knowledge of and attitudes toward hospice care can affect the quality of hospice care. At present, China’s hospice care institutions are suffering from an obvious shortage of nursing staff. Since clinical nurses are the main force behind the future provision of hospice care, their knowledge of, attitudes and willingness to practice can greatly promoted the growth of hospice care, however, available data on clinical nurses’ willingness to practice hospice care are limited.

**Methods:**

A cross-sectional descriptive study design was employed to collect data from 1833 nurses working in tertiary or secondary general hospitals in Guangxi, China. We examined nurses’ demographic characteristics and scores on the Chinese version of the hospice care knowledge scale, the Chinese version of the Bradley Attitude Assessment Questionnaire, and a brief quiz concerning their willingness to practice hospice care in the future. Descriptive, single factor, multiple regression analyses and logistic regression analyses were used for data analysis.

**Results:**

Nurses displayed moderate mean scores for both knowledge of and attitudes, and only 505 (27.5%) nurses expressed their willingness to practice hospice care, 1329 (72.5%) of nurses sampled expressed their unwillingness or uncertainty. Multivariate regression analyses showed that education, professional qualification, monthly income, whether they had been trained in hospice care, and willingness to practice hospice care were the main influencing factors of knowledge; education, whether they lived with someone aged >60 years, and whether they had been trained in hospice care were main factors influencing attitudes. Additionally, logistic regression analyses showed that hospice care knowledge, whether they had been trained in hospice care, and whether they had clinical experience affected the nurses’ willingness to practice hospice care.

**Conclusion:**

This study highlighted a knowledge gap and moderate attitudes toward hospice care among nurses, and most nurses did not prefer to practice hospice care. Having been trained in hospice care was the main common factor of nurses’ knowledge of, attitudes toward, and willingness to practice hospice care in the future, indicating the necessity to provide nurses with more targeted hospice care training.

## Introduction

With the increasing burden of non-communicable diseases and an aging population, the global demand for hospice care is increasing and will likely continue to increase [1]. Hospice care is related to patients’ quality of life, helps to reduce meaningless overtreatment, and results in a reduction of wasted medical resources [2]:“Hospice care is an approach that improves patients’ quality of life and their families facing the problem associated with life-threatening illness, through pain prevention and relief using early identification and impeccable assessment and treatment of pain and other problems, physical, psychosocial and spiritual” [3].

However, in most countries, there is still a large unmet demand for hospice care services [4]. In moderately developed countries, such as China, the demand for effective hospice care services may become even greater. The quality of death index measures the development of hospice care. The "2015 Quality of Death Report" [5] published by The Economist in 2015 evaluated the death index of 80 countries and regions: the United Kingdom ranked 1st, Australia 2nd. The United States 9th, while China ranked 71^st^. The National Bureau of Statistics of China released a population data on February 28, 2019, indicated that the number of people aged ≥ 60 years was 254 million, accounting for 18.1% of the total population. Among them, the number of people aged ≥65 years in Guangxi was 4.96 million [6, 7], however, according to statistics released in 2019, as of the end of 2018, there were 2,342 medical institutions with hospice care departments in China, including 259 tertiary hospitals and 469 secondary hospitals [8], Thus, the coverage rate of hospice care is only 1% [9]. This highlights the unmet need for patients and elderly, while there is also a shortage of preparedness among hospice care professionals [2].

Therefore, the "China Nursing Career Development Plan (2016–2020)" proposes to promote, strengthen and develop hospice care [10]. In 2016, the Central Committee of the Communist Party of China and the State Council issued the "Healthy China 2030" Planning Outline, which clearly stated that national health is the fundamental purpose of building a healthy China, and we must try our best to achieve full health services and health protection from the fetus to the end of life. In February 2017, the National Health and Family Planning Commission issued the "Basic Standards and Management Norms for hospice care Centers (Trial)" and "Practice Guidelines for Hospice Care (Trial)" to guide various institutions to develop the hospice care [11]. To date, China’s hospice care services has made great progress, however, its development is still hindered by factors such as imperfect system, low public awareness, and nursing staff shortages [12, 13]. Nurses play an essential role in the work of hospice care, they allocate more of their time to patients and families than those in other disciplines [14, 15], and their knowledge and attitudes toward hospice care can affect its quality [16, 17]. Robbins’ motivational theory also notes that employees who are willing to engage in a certain industry can set reasonable and clear goals, increase their sense of self-efficacy, and improve work quality and efficiency [18]. Therefore, since clinical nurses are the main force behind the future provision of hospice care, their knowledge, attitudes and willingness to practice hospice care can greatly promoted the growth of hospice care.

Guangxi province is located on the southern coast of China and is a Zhuang Autonomous Region. The development of hospice care has been slow. A study [19] conducted in Guangxi province in 2019 showed that 1200 undergraduate nursing students expressed their knowledge about palliative care is minimal with the majority holding negative attitudes and being unwilling to work in palliative care in the future. To our knowledge, the previous studies were main interested in the knowledge and attitudes of nursing students and nurses in different provinces [20, 21], however, there are few openly accessible studies concerning nurses’ knowledge and attitudes toward hospice care in Guangxi. Moreover, this novel study investigated nurses’ willingness to practice hospice care and analyzed its influencing factors. The findings bridge a key gap in this area and serve as baseline data for developing hospice care training programs in Guangxi.

## Materials and methods

### Study design and sample size

This was a cross-sectional descriptive study that employed convenience sampling. There are 40 tertiary public hospitals and 128 secondary public hospitals in Guangxi. The required sample size was determined by using a formula required for the cross-sectional surveys. The prevalence rates of nurses’ knowledge of, attitudes toward, and willingness to practice hospice care are unknown. Therefore, we took the proportion to be 50% (*p* = .5) to recruit a larger sample size. By using a margin of error of 5%, the Deff of 2, adding a non-response rate of 10%, the minimum sample size required was 820.

### Participants and period

Nurses employed at hospitals in Guangxi province in China were recruited for the study. The inclusion criteria were as follows: nurses from Guangxi who worked at tertiary or secondary public hospitals had obtained a nurse practitioner qualification certificate and volunteered to participate. From October to December 2020, 1833 nurses were invited to participate.

### Measurements

According to literature review and experts consultation, self-made general information and demographic questionnaires, including sex, age, ethnicity, education, professional qualifications, working years, monthly income, marital status, religion, whether the participants are the only child in their families, whether there is a family member with a major illness, whether they lived with someone older than 60 years, whether have experienced the death of a family member or friend, whether they have experience caring for a dying patient, whether they have been trained in hospice care.

The Hospice Care Knowledge Scale was compiled with reference to the relevant knowledge in the book "Hospice care and palliative care" published by Meng [22]. It has been widely used by many scholars in China to assess clinicians’ knowledge of hospice care [23, 24], The scale had good reliability and validity in our pretest, which was completed by 80 nurses: Cronbach’s ɑ value was 0.936, and the content validity was 0.860. The scale comprises 16 items with three possible responses (true, false, not sure). Answers were coded as follows: 1 = "true", 0 = "false" or "not sure". The scale addresses four categories: (1) the basic concepts and content of hospice care, (2) the target patients, (3) the basic philosophy and principles, and (4) the use of pain medication. Total score range of 0–16 points, and higher scores indicate greater knowledge about hospice care.

The Bradley Attitude Assessment Questionnaire was developed by Yale University School of Medicine, USA and is a simple clinical tool to assess the attitudes of medical staff towards hospice care [25]. Chinese scholar Zou Min [26] introduced the tool and conducted cultural commissioning. The Chinese version of the Bradley Attitude Assessment Questionnaire has good reliability and validity [27]. In this study, the Cronbach’s ɑ value was 0.836, and the content validity was 0.860. The 12-item questionnaire comprises three dimensions: professional responsibilities and roles, hospice care effectiveness and nurse-patient communication. Responses are made using a five-point scale from one “strongly disagree” to five “strongly agree.” Total scores range 12–60 points, and higher scores indicate better attitudes toward hospice care among the nursing staff.

Participants reflected their willingness to practice hospice care by answering the following questions: “Would you like to practice hospice care in the future?” Available responses were yes, no, and not sure. If they chose no or not sure, then they were asked to explain the reasons (more than one answer was possible); for example, low pay, being overworked, afraid to face death, sad working environment, and so on.

### Data collection and ethical considerations

The online survey (via the questionnaire website platform) was sent to the heads of each hospital who were then asked to send it to nurses. The survey included an invitation letter containing information regarding the purpose, study procedures, and the time required to complete the questionnaire (10–20 minutes). Consent through an electronic signature was assumed if participants connected to the website link and completed the questionnaire. Participants could complete the questionnaire via a computer or smartphone (through a web link or scanning a quick response code). The study procedures, including the permission to adopt the knowledge and attitude questionnaires, were reviewed, and approved by the Institutional Review Board of The Second Affiliated Hospital of Guangxi Medical University (No.2020-KY0128). Participation was voluntary and participants were informed that they could withdraw from the study at any time. All data collected from the participants are anonymous and confidentiality was ensured to protect the participants’ privacy.

### Data analysis

Data were analyzed using IBM SPSS version 23.0. Frequencies and percentages were used to summarize categorical variables (willingness to practice hospice care in the future), and mean and standard deviation (SD) were used to express continuous variables (knowledge and attitude). Independent samples t-tests were conducted to compare the means between two groups, and analyses of variance (ANOVAs) were used to compare the means for more than two categories. First, potential factors that influence knowledge and attitudes were identified by univariate analyses, such as independent samples t-tests, ANOVAs, and chi-square(χ^2^) tests, to analyze differences between groups. Knowledge and attitudes scores were the dependent variables in multivariate linear regression analysis, and willingness to practice hospice care in the future was the dependent variable in logistic regressions analysis. Next, we performed a multiple linear and logistic regression analysis using all potential factors that were identified earlier by the univariate analyses. The significance level was set at .05 with a 95% confidence interval.

## Results

### Demographics

A total of 1833 nurses participated in the study. 63 were male (3.4%), and 1770 were female (96.6%). The age of the participants ranged from 20 to 58 (31.52±8.10) years, with working experience ranged from 0 to 39 (10.21±8.57) years. Most nurses worked at tertiary hospitals (76.2%), others from the secondary hospitals (23.8%). There were 70.9% nurses had a vocational training degree, only 29.1% nurses had bachelor degree or above. The minority of nurses (17.5%) had received training about hospice care previously, and only 11.2% nurses had hospice care experiences. Table 1 presents other detailed demographics.

**Table 1:** Univariate analysis of knowledge and attitudes of different demographic characteristics of nurses (N = 1833).

item	n	Knowledge				Attitudes			
		M(SD)	t/F		*P*	M(SD)	t/F		*P*
Hospital			0.836		.403		-0.408		.683
Tertiary	1397	8.93(2.22)				34.86(3.58)			
Secondary	436	8.73(2.12)				34.94(3.36)			
Sex			0.923		.356		-0.679		.497
Female	1770	8.90(2.24)				34.87(3.50)			
Male	63	8.38(2.96)				35.17(4.45)			
Whether the participants are the only child			0.879		.380		-1.206		.228
Yes	160	9.17(3.09)				34.56(3.89)			
No	1673	8.86(2.81)				34.91(3.49)			
Nationality			1.675		.188		1.020		.361
Han	1022	8.99(2.24)				34.77(3.50)			
Zhuang	727	8.81(2.37)				35.02(3.60)			
Others	84	8.15(2.47)				34.90(3.35)			
Education			-7.730		< .001		5.132		< .001
vocational training degree	1299	8.38(2.47)				35.16(3.40)			
≥ Bachelor’s degree	534	10.08(3.31)				34.20(3.74)			
Professional qualification			38.228		< .001		4.071		.017
Primary	1230	8.35(2.49)				34.93(3.47)			
Middle	481	9.64(3.18)				34.97(3.64)			
High	122	11.37(2.78)				34.00(3.64)			
Monthly income			23.673		< .001		1.331		.183
<5000 CNY^a^	968	8.29(2.58)				35.01(3.42)			
5000–10000 CNY^a^	836	9.49(3.11)				34.79(3.67)			
≥10000 CNY^a^	29	10.87(3.22)				34.98(4.01)			
Marital status			2.454	.201			3.211	.241	
Unmarried	569	8.84(2.31)				34.42(3.60)			
Married	988	9.13(2.98)				34.11(3.52)			
Divorced or widowed	276	9.34(3.10)				34.54(3.68)			
Religious beliefs			1.347		.178		-1.688		.091
Yes	52	8.90(2.35)				35.69(3.94)			
No	1781	8.08(2.53)				34.85(3.92)			
Do you have a family member with an illness?			2.552		.011		0.775		.438
Yes	311	9.45(3.10)				34.85(3.53)			
No	1522	8.76(2.38)							
Do you have lived with someone aged >60 years?			-0.371		.711		2.565		.010
Yes	1131	8.85(2.40)				35.04(3.49)			
No	702	8.927(2.28)				34.61(3.59)			
In the past year, have you experienced the death of a relative or friend?			-0.910		.363		-0.13		.891
Yes	518	8.82(2.38)				34.90(3.49)			
No	1315	9.02(2.30)				34.87(3.55)			
Have you been trained in hospice care?			-6.210		< .001		4.891		< .001
Yes	320	10.24(3.71)				35.06(3.46)			
No	1513	8.59(2.32)				34.01(3.75)			
Do you have any hospice care experience?			-5.581		< .001		3.357		.001
Yes	206	10.54(3.46)				34.98(3.48)			
No	1627	8.67(2.31)				34.10(3.81)			
Would like to practice hospice care			7.258		< .001		-2.763		.006
Yes	505	10.06(3.03)				34.51(4.01)			
No or unsure	1328	8.43(2.46)				35.02(3.31)			

^a^CNY, China Yuan.

### Knowledge about hospice care and influencing factors

Participants’ mean score was 10.97 (SD = 2.52) out of 16. Questions about the basic concepts and content of hospice care were most correctly answered (84.0% and 89.0%, respectively). While the questions about the use of pain medication dimension were poorly answered (24%). In Table 1, the univariate analysis findings showed that significant differences occurred in nurses’ hospice care knowledge scores owing to education, professional qualification, monthly income, whether a family member has an illness, whether they have been trained in hospice care, whether they have hospice care experience, and their willingness to practice it. However, multiple linear regression analysis showed that education, professional qualification, monthly income, and willingness to practice hospice care were influencing factors in nurses’ hospice care knowledge (Table 2).

**Table 2:** The results of the multiple linear regression analysis of the influencing factors of nurses’ hospice care knowledge.

Independent variable	B	SE	β	*t*	*P*
Constant	5.837	0.871	-	6.701	< .001
Education	1.210	0.221	0.126	5.475	< .001
Qualification titles	0.930	0.172	0.130	5.412	< .001
Monthly income	0.479	0.196	0.058	2.440	.015
Whether they have been trained in hospice care	0.889	0.279	0.077	3.185	.001
whether they have hospice care experience	0.966	0.334	0.070	2.891	.004
Would they would like to practice hospice care	-1.213	0.222	-0.124	-5.476	< .001

95%CI, R^2^ = 0.100, F = 28.908, P < .001.

### Attitudes about hospice care and influencing factors

Nurses’ mean scores on the Chinese vision of Bradley Attitude Assessment Questionnaire score was 34.84 (SD = 3.53) out of 60, They scored the highest on the care effectiveness dimension (mean = 14.63, SD = 1.82), followed by professional responsibilities and roles (mean = 10.54, SD = 2.56) and nurse-patient communication (mean = 9.71, SD = 1.67; Table 3), As shown in Table 1, the univariate analysis revealed that significant differences occurred in nurses’ hospice care attitudes scores owing to education, professional qualification, whether they lived with some older than 60 years, whether they had been trained in hospice care, whether they had any hospice care experience, and their willingness to practice hospice care. However, multiple linear regression analyses showed that education, whether they lived with some older than 60 years, and whether they had been trained in hospice care were the influencing factors of nurses’ attitudes toward hospice care (Table 4).

**Table 3:** Nurses’ attitudes toward hospice care (N = 1833).

Category	Number of questions	Total scores M(SD)	Single scores M(SD)
Professional responsibilities and roles	4	10.54 (2.56)	2.64 (0.64)
hospice care effectiveness	5	14.63 (1.82)	2.92 (0.36)
Nurse-patient communication	3	9.71 (1.67)	3.24 (0.56)
*F*	486.584		
*P*	< .001		

**Table 4:** The results of the multiple linear regression analysis of the influencing factors of nurses’ hospice care attitudes.

Independent variable	B	SE	β	*t*	*P*
Constant	37.541	0.619	-	60.693	< .001
Education	-0.887	0.183	-0.114	-4.559	< .001
whether they lived with someone older than 60 years	-0.436	0.168	-0.060	-2.602	.009
Whether they had been trained in hospice care	-0.771	0.234	-0.083	-3.294	.001

95%CI, R^2^ = 0.033, F = 10.463, P < .001.

### Willingness to practice hospice care in the future and its influencing factors

**Fig 1:**
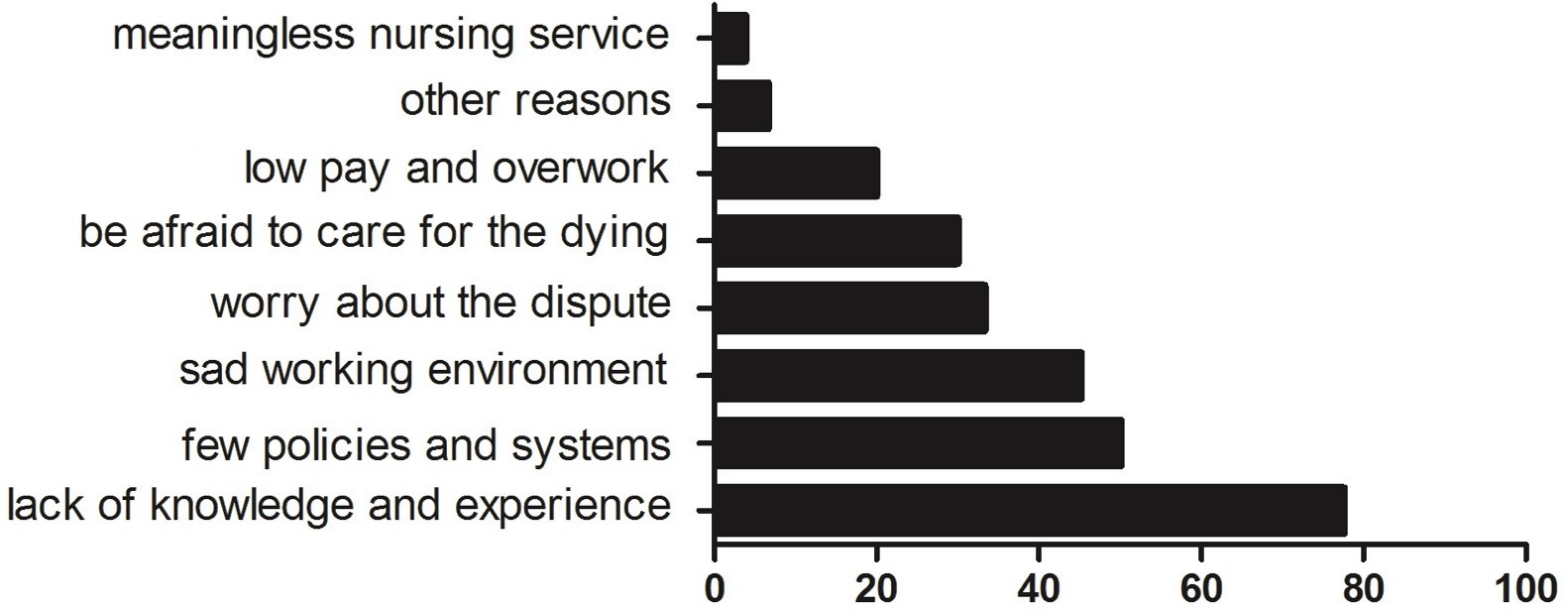
The reasons for unwillingness or uncertainty to practice hospice care (%).

About one-quarter (n = 505, 27.56%) of the participating nurses expressed their willingness to practice hospice care, while 268 (14.6%) were unwilling to practice it and 1061 (57.9%) were unsure (Table 5). The main reasons for unwillingness or uncertainty to practice hospice care are showed in Fig 1. Only a few participants chose “other reasons”, the most cited reasons were that their family members do not support it and they were satisfied with their current job.

**Table 5:** Univariate analysis of willingness to practice hospice care in the future of different demographic characteristics of nurses (N = 1833).

item	n	willingness to practice hospice care				
		Yes (n%)	No or Not sure (n%)	*x* ^2^	*P*	
Hospital				1.254	.263	
Tertiary	1397	394(21.5)	1003(54.1)			
Secondary	436	111(6.1)	325(17.3)			
Sex				0.222	.637	
Female	1770	486(26.5)	1284(70.0)			
Male	63	19(1.0)	44(2.4)			
Whether the participants are the only child				0.029	.865	
Yes	160	45(2.5)	115(6.3)			
No	1673	460(25.1)	1213(66.6)			
Nationality				6.131	.047	
Han	1022	278(15.2)	744(40.6)			
Zhuang	727	194(10.6)	533(29.1)			
Others	84	33(1.8)	51(2.8)			
Education				1.567	.211	
vocational training degree	1299	347(18.9)	952(51.9)			
≥ Bachelor’s degree	534	158(8.6)	376(20.5)			
Professional qualification				13.258	.001	
Primary	1230	310(16.9)	920(50.2)			
Middle	481	148(8.1)	333(18.2)			
High	122	47(2.6)	75(4.1)			
Monthly income				8.034	.018	
<5000 CNY^a^	968	240(13.1)	728(39.7)			
5000–10000 CNY^a^	836	255(13.9)	581(31.7)			
≥10000 CNY^a^	29	10(0.5)	19(1.0)			
Marital status				37.367		< .001
Unmarried	569	105(5.8)	464(25.3)			
Married	988	301(16.4)	687(37.5)			
Divorced or widowed	276	99(5.4)	177(9.6)			
Religious beliefs				1.338	.247	
Yes	52	18(0.9)	34(1.9)			
No	1781	487(26.6)	1294(70.1)			
Do you have a family member with an illness?				3.441	.064	
Yes	311	99(5.4)	212(11.6)			
No	1522	406(22.1)	1116(60.9)			
Do you lived with someone age >60 years?				0.134	.714	
Yes	1131	315(17.2)	816(44.6)			
No	702	190(10.4)	512(27.9)			
In the past year, have you experienced the death of a relative or friend?				2.752	.097	
Yes	518	157(8.6)	361(19.7)			
No	1315	348(19.0)	967(52.8)			
Have you been trained in hospice care?				99.593	< .001	
Yes	320	143(7.8)	177(9.7)			
No	1513	362(19.7)	1151(62.8)			
Do you have any hospice care experience?				34.038	< .001	
Yes	206	92(5.0)	114(6.2)			
No	1627	413(22.5)	1214(66.2)			

^a^CNY, China Yuan.

The willingness of nurses to practice hospice care was divided into two types: willing, unwilling/uncertain. The logistic regression model demonstrated that nurses’ knowledge of hospice care and whether they had been trained in it affected their willingness to practice hospice care (Table 6).

**Table 6:** The results of logistic regression analysis concerning the influencing factors of nurses’ willingness to practice hospice care.

Independent variable	B	SE	Wald *x*^2^	*P*	OR (95%CI)
knowledge of Hospice care	-0.078	0.014	30.806	< .001	0.903(0.900,0.951)
whether they have been trained in hospice care	-0.698	0.142	24.157	< .001	0.498(0.377,0.657)
whether they have hospice care experience	-0.388	0.168	5.302	.021	0.678(0.488,0944)

## Discussion

Per the "Healthy China" grand strategy, aging and chronic diseases bring challenges to China’s health system. Although China has made real progress to the promotion of hospice care throughout the country, services remain limited [28]. Previous studies have highlighted gaps in knowledge and indicated that nurses only hold moderately positive attitudes toward hospice care [29–31]. We found that nurses’ mean knowledge score was 10.97(SD = 2.52), which was higher than the scores displayed in previous studies conducted in other provinces in China [32, 33]. The multiple linear regression analyses confirmed that several factors can influence nurses’ knowledge about hospice care: nurses with further education, higher professional qualifications and who were willing to practice hospice care comparatively reported the highest knowledge scores.

There is no denying that the nurses with higher educational backgrounds receive a more comprehensive and complete education, including courses related to hospice care. Consistently, Abudari and colleagues [34] found that nurses with a higher degree had more knowledge of palliative care than did their less-educated counterparts, which may be owing to the amount of palliative care education in the nursing curriculum in Saudi Arabia. Additionally, nurses with higher professional qualifications are also older, have a good monthly income, have more clinical experience, have more continuing education and training opportunities, and have acquired more knowledge about hospice care than their counterparts. Previous studies indicated that nurses’ level of education and working years indicated significant association with better knowledge scores [35, 36]. Furthermore, if nurses expressed their willingness to practice hospice care, they may be more willing to take the initiative to learn knowledge about hospice care, so they have a better understanding and mastery of hospice care knowledge.

In the present study, the nurses reported moderate attitudes toward hospice care, which was consistent with previous studies conducted in other countries, including Turkey [37], India [38] and Ethiopia [39]. Among the mean scores of three dimensions of the attitude questionnaire, nurse-patient communication received the highest mean score, as nurses have direct and frequent communication with the patients [40]. Thus, they may have relevant experience communicating with dying patients.

The multiple linear regression analyses suggested that education, whether they have been trained in hospice care, and whether they live with someone older than 60 years can influence nurses’ attitudes toward hospice care. The attitude scores of nurses with vocational training degree were lower than those of nurses with a bachelor’s degree or beyond, which coincides with the results of previous studies from China [21, 41] and Australia [42]. Unsurprisingly, nurses with more education and higher hospice care knowledge scores hold more positive attitudes about hospice care than their counterparts [43].

Moreover, nurses who do not live with someone older than 60 years had low attitude scores. If there are elderly people in the family, the nurses may feel and understand the relatives’ expectations of spending the rest of their lives peacefully, thus, fostering the importance of hospice care work. Nurses who do not live with an older adult may not share this same perspective. Shi and colleagues [44] found that caregivers who had experienced the death of a relative or friend held more positive attitudes toward caring for the dying. Moreover, nursing staff who had been trained in hospice care displayed a more positive attitude toward it than those who had not, which was aligned with prior results of Etafa [45] and Farman [36]. Since education or training can bring behavioral change, related departments should provide high-quality and standardized continuity training concerning hospice care based on the current accumulated experience in China to raise awareness about it in Guangxi. This could foster nurses’ passion for providing hospice care.

Among the participating nurses, only about one-quarter were willing to practice hospice care, while the majority were unwilling or hesitant. The three main reasons for nurses’ unwillingness or uncertainty were the lack of knowledge and experience related to hospice care (77.59%), few palliative policies and systems (50.10%), and that it is a sad working environment (45.21%). The least common was that the service was meaningless. Logistic regression analyses highlighted that nurses’ knowledge, whether they had been trained in hospice care, and whether they had clinical experience in hospice care were the influencing factors of nurse’ willingness to practice hospice care.

To date, the establishment of hospice care services in medical institutions throughout Guangxi is lacking, the service capacity is insufficient, and an independent medical education discipline and a systematic clinical professional system have not been formed [19, 46]. In 2017, the Guangxi local standard of "Service Standards for hospice care for Elderly Care Institutions" was released and implemented, filling the gap in the service standards of elderly care institutions. In December 2019, the department launched the second batch of hospice care pilot cities, and Qinzhou Guangxi was included [47], which enabled further exploration and development of hospice care. Regarding recruiting a nursing team for hospice care, Deng [48] proposed that senior nurses with experience in caring for dying patients should take the lead in participating in the construction of hospice care to promote its development. Concurrently, nurses should complete continuing education and training to increase their professional knowledge of hospice care, develop relevant communication skills and knowledge of psychological counseling, and alleviate their fear of death.

Notably, our survey indicated that the attitudes scores of nurses who were unwilling or unsure of practicing hospice care were higher than nurses who were willing to practicing hospice care. This may be related to the inadequate development hospice care in China, and there is no clear policy orientation concerning practicing hospice care provided for nurses, which leads to a low willingness to practice hospice care. Hence, targeted training programs on nurses concerning hospice care are necessary and measures need to be taken to increase awareness of hospice care among the public. Moreover, it is necessary to incentivise the nurses switch to hospice care, this would in part be facilitated through new welfare policies, and clarify the current ambiguity in the law concerning hospice care, legal protections for and recourses of nurses. While working continuously to ensure that a positive working environment is created to ensure that nursing staff encouraged and motivated to provide hospice care.

### Study strengths and limitations

We employed a cross-sectional design and analyzed the data from a large sample of nurses, which may enhance the generalization of our findings. However, two main limitations should be considered. First, this study was conducted in Guangxi, and there is no additional data from other provinces. Hence, this result cannot be generalized to all Chinese nurses. Second, although the results were valid, there may be other factors that affect nurses’ knowledge of, attitudes toward and willingness to practice hospice care. Consequently, the methodology should be improved and a multicenter investigation should be conducted to enrich our results.

## Conclusion

The nurses in this study displayed insufficient knowledge and moderate attitudes toward hospice care, but overall, their willingness to practice hospice care was low. Having been trained in hospice care was the main common factor behind nurses’ knowledge of, attitudes toward, and willingness to practice hospice care in the future. Hence, More attention should be paid to clinical nurses and nursing students to strengthen their hospice care education and training. Our findings also suggested to improve indigenous policies, systems, and laws to promote hospice care and encourage more nurses to devote themselves to the development of hospice care with full enthusiasm.
